# Infection-induced changes in gene expression linked to foraging choices in self-medicating *Lasius platythorax* ants

**DOI:** 10.1098/rspb.2026.2357

**Published:** 2026-11-11

**Authors:** Jason Rissanen, Christoph Hahn, Heikki Helantera, Dalial Freitak

**Affiliations:** ^1^ Institute of Biology, Karl Franzens University of Graz, Graz, Styria, Austria; ^2^ Tvärminne Zoological Station, University of Helsinki, Hanko, Uusimaa, Finland; ^3^ University of Oulu, Oulu, Finland

**Keywords:** taste, self-medication, infection, *Malvolio*, foraging, sensory perception

## Abstract

Many animals self-medicate by altering their diet intake to include various compounds in response to a pathogen infection, but little is known about the underlying mechanisms that drive the behaviour. To gain a deeper understanding of self-medication, we used a combined approach of behavioural and physiological methods in *Lasius platythorax*, a species of ants in which this behaviour has not been reported before. By giving colonies a diet choice between a standard food and a medicinal one containing H_2_O_2_, we show that colonies infected with a fungal pathogen increase their preference towards the medicinal food which healthy colonies avoid and is associated with improved survival in infected colonies. Differential gene expression analyses revealed significant changes in gene expression after pathogen infection and identified *Malvolio*, which is a gene linked to both immune responses and changes in taste perception in other insects. Furthermore, temporal patterns of *Malvolio* expression levels, monitored daily throughout a prolonged infection trial, showed a strong correlation with foraging frequency on medicinal food, which further supports *Malvolio*'s important role in self-medication. Our results not only confirm self-medication in *L. platythorax* but also give fundamental insights into how infection-induced changes in sensory pathways could drive self-medication responses.

## 1. Introduction

Self-medication is an adaptive behavioural response of animals to use biologically active compounds for treatment or prevention of pathogen infections. These compounds can be used through proximity, topical application or diet, of which the latter is the form that has been studied the most [1–5]. What sets self-medication apart from other dietary behaviours is that consuming medicinal compounds is detrimental for healthy animals but beneficial in the case of a pathogen infection [1,2,6]. Many of the compounds used medicinally have toxic properties, and prior studies show that healthy individuals avoid them but increase their consumption in response to pathogen threat or infection [6–10].

This type of dietary flexibility is a key factor that enables animals to overcome the negative consequences of pathogen infections. Dietary preferences do not only affect several life history traits, such as growth and reproduction [11,12], but the ability to modulate diet may also directly impact the capacity to cope with pathogens through affecting immune responses [13–15] or as medicine. Animals use their sensory pathways for assessing information about the nutritional composition and quality of food from multiple sources and regulate their intake to obtain an optimal mixture of nutrients [16,17], including medicinal compounds [7–10]. Medicinal, and toxic, compounds are often characterized as bitter [4,5,8,9,18], which raises the question of whether diseases induce changes in sensory perception that increase foraging on them when infected compared to a healthy state. Some genes have been identified to play a part in innate immunity in insects, which also influence feeding preferences [19–21] and such may have pleiotropic functions that impact both immune responses and sensory pathways to guide dietary behaviours to maintain an optimal diet, as well as the regulation of the intake of medicinal compounds during pathogen infections.

While altering dietary behaviours to include medicine is relatively straightforward for solitary animals, social insects like ants need to respond to the needs of the entire colony. Sociality and large colonies have been integral to the success of ants [22] but make them more susceptible to contracting and spreading disease [23,24]. In the case of a pathogen outbreak, foraging workers may have to rely on information from their nest-mates to regulate the collective intake of nutrition to include medicinal compounds [17,25]. Ants have been shown before to self-medicate by increasing foraging on H_2_O_2_ (a reactive oxygen species, ROS), a bitter compound they normally avoid, or by changing their dietary ratios of protein to carbohydrates in order to treat themselves against fungal and viral pathogens [8–10,26]. Furthermore, *Formica fusca* ants alter their dietary choices on foods containing aphids during the acute phase of a fungal infection, showing that infection does not lead to the loss of discrimination between different food sources but rather implies changes to it during the progression of infection [27]. While it is clear that ants, like other animals, use medication behaviours to cope with pathogen outbreaks, nothing is known about how the interactions between pathogens and hosts drive this behaviour.

Here we study the self-medication response in the ant *Lasius platythorax*, using a diet choice experiment including a standard food with and without an H_2_O_2_ supplement (referred to as ROS food), which has been shown to be medicinal for *F. fusca* and *Solenopsis invicta* [8–10]. To understand how ants react to a pathogen infection on a physiological level as well as potential changes in taste perception during self-medication, we analysed the transcriptome of the ants during infection progression by the generalist entomopathogenic fungus *Beauveria bassiana*. Differential expression analyses allowed us to identify specific genes of interest which we studied in detail using RT-qPCR in a follow-up experiment to see whether the dynamics of gene expression correlate with changes in foraging behaviour. This allowed us to gain first insights into how medication behaviours could be mechanistically influenced by the activity of specific genes in biological pathways that are active during the infection, and whether it could be linked to foraging choices and activity.

## 2. Material and methods

### (a) Experiment A: self-medication

#### (i) Experimental colonies

We collected workers from 15 wild *L. platythorax* nests from a wood clearing in southern Finland (59°54′46.9″ N, 23°15′53.8″ E) in May of 2024. *Lasius platythorax* is a common woodland species in the Euro-Siberian range, with colony sizes ranging from thousands to tens of thousands of workers and a single queen [28]. We minimized the time the colony spent without the presence of a queen prior to the experiment by collecting workers on the morning of the first day of the experiment. From each nest, we created six sub-colonies, one for each treatment combination (treatment: sham-treated/infected; diet: standard/food choice/ROS), for a total of 90 experimental colonies, each with 120 workers.

As experimental nests, we used plastic containers (8 × 15 × 10 cm) with a plaster cast bottom. A circular indentation (3 cm Ø) was made in one end of the plaster to function as a nesting cavity, and a small piece of ceramic tile (4 × 4 cm) was placed on top for cover. The walls of the nest-boxes were lined with a 20% w/v mixture of talcum powder and ethanol to prevent ants from escaping [29]. Each nest had a plastic lid with a segment cut-off to allow for observation of the foraging area. We provided each nest with a 1.5 ml Eppendorf tube filled with water with a cotton plug for water and moisture.

#### (ii) Diet treatments

As the standard food, we used the Bhatkar & Whitcomb [30] diet based on honey, egg and agar. The ROS food was standard food with a 4% H_2_O_2_ concentration. H_2_O_2_ plays a role in immune signalling in insects and can directly harm pathogens [8,31,32] and has been shown to be used as medicine before in *F. fusca* and *S. invicta* [8–10]. In each diet treatment, the sub-colonies were given two Eppendorf caps filled with food, prepared freshly each day. The standard diet treatment was provided with two caps of standard food. The food choice treatment was provided with one cap of standard food and one cap of ROS food. The ROS diet treatment was given two caps of ROS food. This way, in combination with the infection treatment, we could assess the effect of the diet on the survival of ants, either healthy or suffering from a fungal infection.

#### (iii) Infection treatment

*Beauveria bassiana* was grown on solid malt-peptone agar medium in sterile petri dishes at room temperature in the dark. Spores were collected from three separate plates by pipetting 10 ml of 0.05% Triton-X solution on top of the fungus, after which the surface was carefully rubbed with a sterile glass triangle to loosen the spores from the fungus. The spore solutions from all plates were then combined and diluted to the desired 1 × 10^8^ concentration in 0.05% Triton-X.

The sub-colonies assigned to infection treatment were exposed to the spore solution by collecting the ants into a hollow tube with a mesh bottom and a screw-topped lid. The entire tube was then submerged in the spore solution for 5 s while moving the tube inside the liquid to ensure that all the ants in the tube were exposed to the spores. After submersion, the ants were removed from the tube onto absorbent tissue paper and allowed to dry off before returning them into the nest-box. The sub-colonies assigned to the control treatment were sham-treated using the same protocol but were instead submerged in clean 0.05% Triton-X solution.

#### (iv) Observations

The foraging frequency and the food choices were observed by taking pictures of the foraging arena in the nest-boxes of the food choice treatment four times a day (at 10, 14, 18 and 22 to represent foraging throughout the day) for a total of 9 days. An ant was considered a forager if it was in contact with the food with a leg or antennae, as the process of the camera appearing above the nest-box could have startled the ant and interrupted feeding. This method of observation has been used before in studies using a similar set-up [9,27].

Survival was monitored daily in the mornings at 10.00 during the change of the food caps in each nest-box. All dead ants were removed and placed on 0.5% water-agar plates and subsequently incubated at room temperature in the dark for 24 h to confirm that the ants died of the fungal infection.

#### (v) RNA extraction and sequencing

We took samples of ants (10 pooled heads from each nest-box) from the food choice sub-colonies at two different time points: day 2 post-treatment and day 4 post-treatment. The samples were stored in a 1.5 ml Eppendorf tube with 200 µl of RNA Later. The samples were stored at +4°C for the first 24 h, after which they were placed in −20°C for the next 24 h before final storage in −80°C. The sampling dates were chosen to correspond with early-stage infection on day 2, when the spores have penetrated the cuticle and start growing within the host, and acute infection on day 4, after which mortality by the fungus is typically observed [33].

RNA was extracted and isolated using a standard TRIzol (Invitrogen) method according to the manufacturer's protocol, with a final suspension in 40 µl of nuclease-free H_2_O. Sample quality was assessed with a NanoDrop, and lower-quality samples were purified using the GeneJET RNA Cleanup and Concentration Micro Kit (Thermo Scientific) according to the manufacturers' protocol with a final elution of purified RNA in 40 µl of clean Milli-Q H_2_O. We chose to use samples from 10 sub-colonies, 5 exposed to pathogens and 5 controls, representing 5 original nests. Each sub-colony has day 2 and day 4 samples for a total of 20 samples. The samples were sent for sequencing to Novogene (Munich, Germany) and were sequenced on the Illumina NovaSeq 6000 sequencing platform.

### (b) Experiment B: correlation between foraging activity and expression of *Malvolio*

#### (i) Experimental colonies and foraging observations

We collected workers from 15 wild *L. platythorax* nests in May of 2025 in the same area as the year before. Each nest was split into two sub-colonies of 500 workers, each to be used in two different infection treatments (sham-treated/infected), both of which were given the food choice of standard food and ROS food as described in experiment A. Bigger fragments were used in this experiment owing to the necessity of sampling a larger number of ants for qPCR analysis. Infections were conducted using the same protocol as in experiment A but using a spore concentration of 3 × 10^8^ to ensure a higher infection rate in the colonies. We monitored foraging for 13 days after exposure using the same protocol as defined in experiment A. Dead ants were removed daily from the colonies.

#### (ii) RNA extraction and qPCR analysis

We sampled every sub-colony daily by pooling 10 heads from workers randomly collected from the nest-boxes into a 1.5 ml Eppendorf tube with 200 µl RNA. Later RNA was extracted from the samples using the same TRIzol (Invitrogen) protocol as in experiment A. Any remnant DNA was digested using the DNase I kit (Thermo Scientific) and subsequently purified with the GeneJET RNA Cleanup and Concentration Micro Kit (Thermo Scientific). All samples were standardized to contain 56.8 ng µl^−1^, which was the lowest RNA content in the series of samples. The purified RNA was translated into cDNA using the RevertAid First Strand cDNA Synthesis Kit (Thermo Scientific). The 20 µl volume of PCR products in the wells was diluted to a final volume of 100 µl with nuclease-free water. In all the steps with the kits, we followed the protocols provided by the manufacturers.

The RNA sequencing identified three isoforms of the gene *Malvolio*, which we chose as a gene of interest owing to it being part of immune responses and modulation of taste preferences. As the sequences of the isoforms were almost identical, and there being no prior knowledge of whether the isoforms are functionally different, we designed a generalized primer for the *Malvolio* gene (MalG). Actin (Act) was used as a housekeeping gene in the analysis (sequences in supplementary material, table S7).

We used real-time quantitative polymerase chain reaction (RT-qPCR, CFX384 Real-Time System (Bio-Rad)) to analyse the expression of *Malvolio* in 384-well hard-shelled PCR plates. The samples were analysed with the iTaq Universal SYBR Green Supermix (Bio-Rad) with a final volume of 10 µl per well [6 µl master mix: 5 µl SYBR + 1 µl reverse/forward primer mix, 4 µl of sample]. Each sample was run in two technical replicates using a PCR programme of (95°C 30 s; 40 × 95°C 5 s, 60°C 30 s; 95°C 10 s).

#### (iii) Data analysis

All data analysis was done using the R software v.4.5.2 [34], and all graphs were drawn using *ggplot2* [35], with the exception of the upset plot and NMDS plots, which were drawn using the *ComplexHeatmap* [36] and *vegan* [37] packages, respectively.

### (c) Experiment A: self-medication

#### (i) Foraging analysis

The foraging data from the food choice sub-colonies were analysed using a negative binomial generalized linear mixed model from the package *glmmTMB* [38]. The number of foragers was used as the response variable, with day and the interaction between food type (standard/ROS) and treatment (control/exposed) as the explanatory variables. The original nest ID as well as the sub-colony ID nested in time were used as random factors to take nest variation and pseudoreplication into account. Full model: Foragers ∼ Food type × Treatment + (1 | Nest) + (1 | Time/Sub-colony). Model diagnostics were evaluated using the package *DHARMa* [39] and a Durbin–Watson test was conducted to confirm there was no temporal autocorrelation detected in the model (*p* = 0.141). Post hoc tests on the effect of the fixed factors and their interactions were determined with an ANOVA, and further pairwise comparisons were conducted using the function emmeans from the *emmeans* package [40] with a Tukey's *p*-value adjustment.

#### (ii) Survival analysis

The effect of treatment and diets from all treatment nests were analysed using Cox proportional hazard models built with the coxme function from the *coxme* package [41]. The number of ants dying each day was used as the response variable, and the interaction between food type and treatment as explanatory factor. The original nest ID and colony ID were used as random factors. Full model: (Day, Survival) ∼ Food type × Treatment + (1 | Nest) + (1 | Sub-colony). Post hoc tests on the effect of fixed factors and pairwise comparisons were conducted as described above for the foraging analysis.

#### (iii) RNA sequencing analysis

The raw sequencing data were trimmed first using Trim Galore (v.0.6.0; https://github.com/FelixKrueger/TrimGalore), which is a wrapper around Cutadapt [42] and mapped against a reference genome (GenBank accession GCA 964030505.1) of *L. platythorax* [43] using STAR (v.2.7.10b) [44]. Duplicates were removed using the MarkDuplicates (Picard) software (2.18.7; http://broadinstitute.github.io/picard). Read counts per gene were obtained using featureCounts (2.0.6) [45].

The processed data representing a count matrix for all the genes were then analysed for differential gene expression in R using *DESeq2* [46]. We removed all genes with 0 counts for any sample from the analysis, resulting in a comparison of 8589 genes. We built separate models to analyse gene expression for the different time points. For the analysis design, we used the original nest as a batch effect to account for differences between colonies, with treatment (infected/control) as a fixed factor for each model. For the analysis, we used an adjusted *p*-value level of significance of 0.05.

To identify specific biological processes, we performed a GO term enrichment analysis for the differentially expressed genes on the different days using *topGO* [47]. Functional annotation and assignment of GO terms were performed for the full protein complement of *L. platythorax* [43] using InterProScan (5.48–83.0) [48]. As the gene universe for the topGO analysis, we used the list of genes from the gene expression analysis, excluding those with 0 counts (see above). For building the topGO data object, we used biological processes as the ontology category of interest with a node size of 5. Significance tests were conducted using the Fisher statistic with ‘weight01’ as the algorithm, using a *p*-value of 0.05. Functional annotations of individual gene sequences of interest (significantly up-/downregulated and part of a significantly enriched biological process) were verified/refined through BLAST (basic local alignment search tool) against the UniProtKB/Swiss-Prot database using the NCBI web interface (https://blast.ncbi.nlm.nih.gov/Blast.cgi).

### (d) Experiment B: correlation between foraging activity and expression of *Malvolio*

#### (i) Foraging

The foraging data for the correlation between *Malvolio* expression and foraging were analysed with a negative binomial model using the glmmTMB function. The number of foragers used as a response variable, with day, treatment and food type, as well as their interaction, was used as fixed effects. The original nest ID and the sub-colony ID nested in time were used as random factors. Full model: Foragers ∼ Day × Treatment × Food type + (1 | Nest) + (1 | Time/Sub-colony). An ANOVA was used to determine the significance of the fixed effects, and pairwise comparison was conducted using the *emmeans* package with a Tukey's *p*-value adjustment.

On days 6, 8 and 12, the control colonies had no foraging events on ROS food, making formal comparison on those days impossible owing to complete separation in data. To deal with the issue, we added a single foraging data point for those days. The addition of a single foraging data point did not influence the results. A large fraction of colony 7 escaped during the experimental period and was therefore omitted from further analysis.

#### (ii) QPCR analysis

The RT-qPCR gene expression data were analysed and visualized using the *MCMC.qpcr* package [49]. We built a model using the MCMC.qpcr function, where days and treatment as well as their interaction were set as fixed factors, with sub-colony ID and original nest ID added as random factors. As samples were analysed on several qPCR plates, we added the ID of the qPCR plate as a random factor to account for variance caused by different plate runs. Actin was used as a control gene in the model.

## 3. Results

### (a) Experiment A: self-medication

#### (i) Foraging

The presence of ROS in the food significantly affected the foraging choices of the sub-colonies (food type: $\chi^{2}$ = 143.569, *p* < 0.001) with the standard food preferred in both the control (*z* = 9.109, *p* < 0.001) and infected sub-colonies (*z* = 8.172, *p* < 0.001). Exposure to *B. bassiana* did not affect the overall foraging frequency (treatment: $\chi^{2}$ = 0.050, *p* = 0.824), and pairwise comparisons did not show a significant difference in overall foraging frequency between the control and infected sub-colonies on either the standard food (*z* = 0.936, *p* = 0.349) or the ROS food (*z* = −1.890, *p* = 0.059). However, exposure to *B. bassiana* did significantly affect the foraging choices of the sub-colonies on the two foods in the food choice treatment (food type × treatment: $\chi^{2}$ = 4.578, *p* = 0.032) (figure 1A).

**Figure 1.**
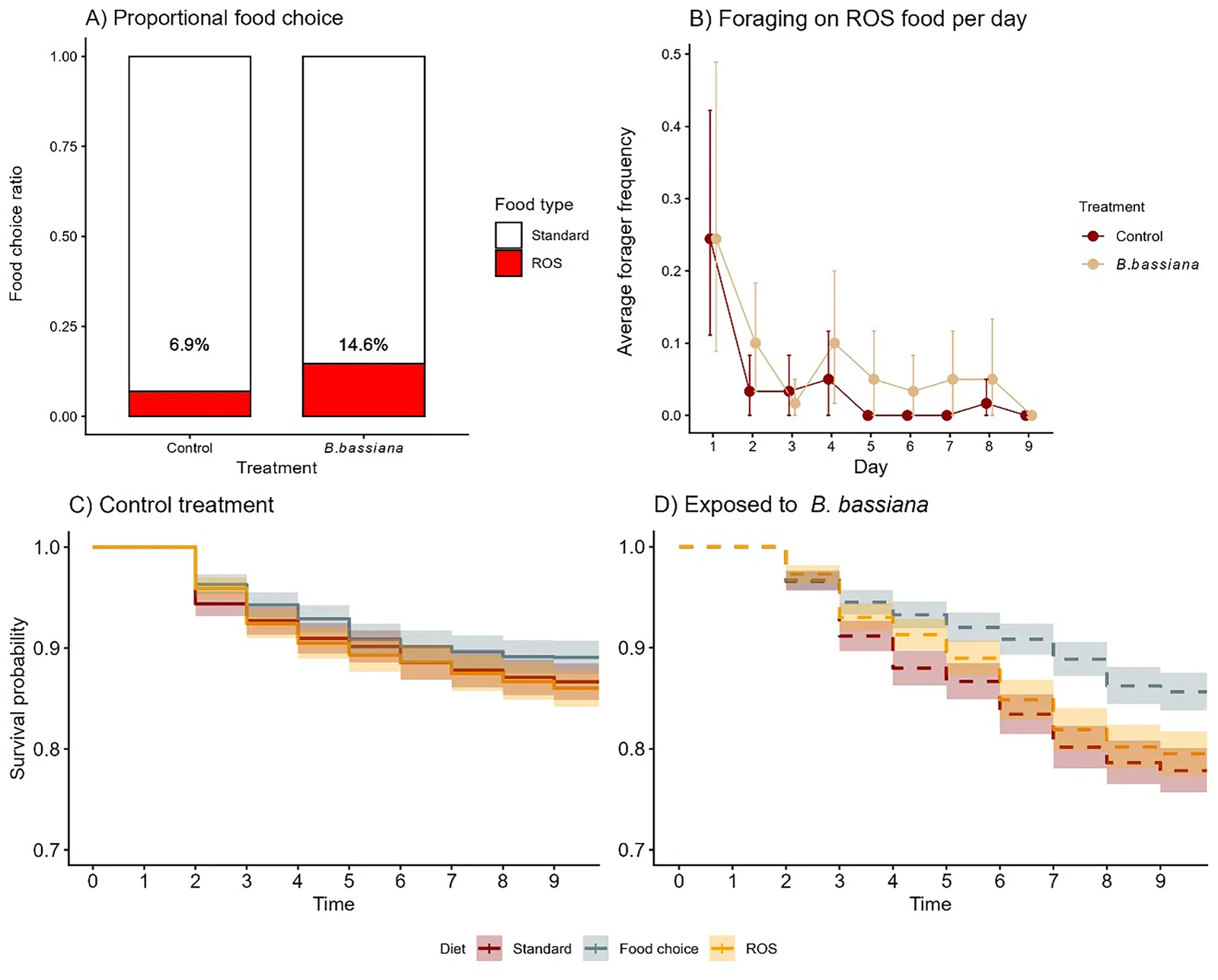
Foraging on different foods in the food choice diet between the two treatments. (A) Infection significantly affected foraging choices between control colonies and those exposed to *B. bassiana* (food type × treatment: $\chi^{2}$ = 4.578, *p* = 0.032) shown as a proportion of foraging frequency on the two foods. Part (B) shows the foraging activity on the ROS food over each day. (C) Survival of *L. platythorax* workers in control colonies was not affected by diet. (D) Survival of *L. platythorax* workers exposed to *B. bassiana* on different diets. Whiskers and shaded areas indicate the 95% confidence intervals. Sample size: (A) and (B) *n* = 30 colonies, (C) and (D) *n* = 8900 ants.

#### (ii) Survival

Infection treatment significantly affected the survival in the sub-colonies (treatment: $\chi^{2}$ = 7.962, d.f. = 1, *p* = 0.005), but diet did not have an overall effect on survival (diet: $\chi^{2}$ = 5.275, d.f. = 2, *p* = 0.071), nor did the interaction of the two (treatment × diet: $\chi^{2}$ = 0.559, d.f. = 2, *p* = 0.756). Within-diet comparisons show that infection by *B. bassiana* significantly increased mortality within the standard diet sub-colonies (*z* = −2.082, *p* = 0.037), but neither within the ROS diet sub-colonies (*z* = −1.724, *p* = 0.085) nor the food choice sub-colonies (*z* = −1.005, *p* = 0.315) (figure 1C).

Within-infection treatment comparisons show that the differences in survival in the control sub-colonies are not significant owing to the different diets (standard vs. food choice: *z* = 0.773, *p* = 0.719; standard vs. ROS: *z* = −0.345, *p* = 0.937; food choice vs. ROS: *z* = −1.119, *p* = 0.502). The differences in the survival of the exposed sub-colonies on different diets were also not significant (standard vs. food choice: *z* = 1.858, *p* = 0.151; standard vs. ROS: *z* = −0.007, *p* = 1; food choice vs. ROS: *z* = −1.839, *p* = 0.157).

#### (iii) RNA sequencing and GO term enrichment

Gene expression was significantly changed by the pathogen infection. Two days after exposure to the pathogen, 504 genes were significantly differentially expressed compared to the control sub-colonies, with 218 upregulated and 286 downregulated in infected ants. On the fourth day post-exposure, the number of differentially expressed genes increased to 946; of them, 399 were upregulated and 557 were downregulated in the infection treatment. Of the upregulated genes, 34 were consistently upregulated during both sampling events, whereas of the downregulated genes, 52 were downregulated in both time points. Four genes that were upregulated on day 2 were downregulated on day 4, and nine genes that were downregulated on day 2 were upregulated on day 4 (figure 2). Full results of the differential gene expression analysis are presented in S2 in the supplementary material.

**Figure 2.**
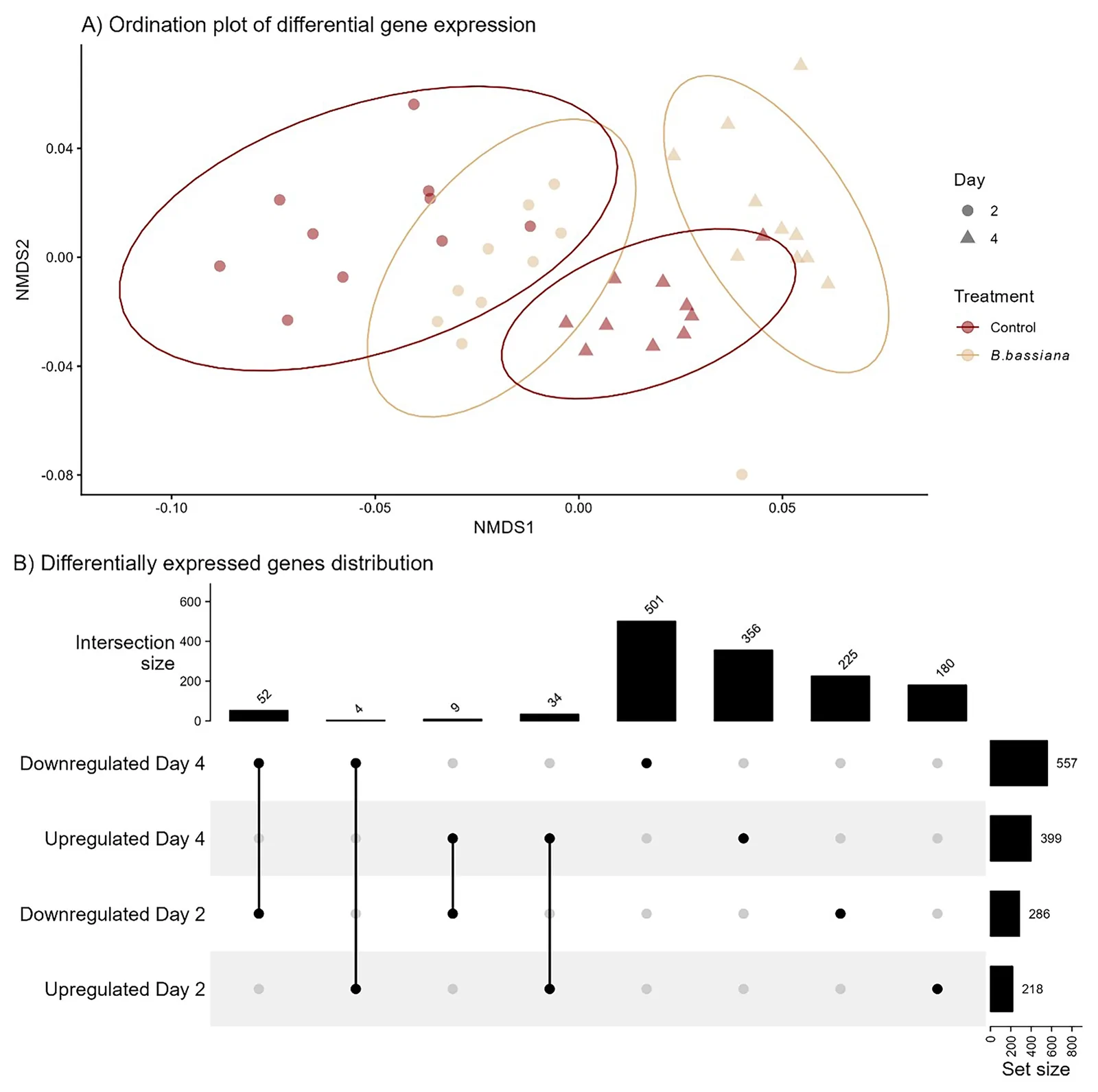
Gene expression results between the treatments over time in experiment A. Part (A) shows an NMDS ordination plot with each symbol representing a sample. The colour represents the infection treatment and the shape of the symbol the sampling day. Part (B) shows an upset plot summarizing counts of significantly up- and downregulated genes on day 2 and, while the majority of the genes were unique to days and treatments, a small number of genes were consistently down- or upregulated on both days 2 and 4 within a treatment.

Results of enrichment analyses of biological process gene ontology (GO) terms were conducted for all time points (supplementary material, tables S1–S4). We identified 49 biological process gene GO terms enriched in the set of downregulated genes on day 2. Forty-nine processes were enriched in the set of upregulated genes during this time. On day 4, 55 biological processes were enriched in the set of downregulated genes. Sixty processes were enriched in the set of upregulated genes on day 4, including processes which are connected to immune functions (arachidonic acid secretion, GO:0050482; manganese iron transmembrane transport, GO:0009987) and sensory development (peripheral nervous system development, GO:0007422; sensory organ development, GO:0007423; salivary gland development, GO:0007431) (figure 3). Our analysis identified one further significantly upregulated process of particular interest: sensory perception of sweet taste (GO:0050916). Within this process, two out of five genes were significantly upregulated and were identified as two isoforms of the *Malvolio* gene.

**Figure 3.**
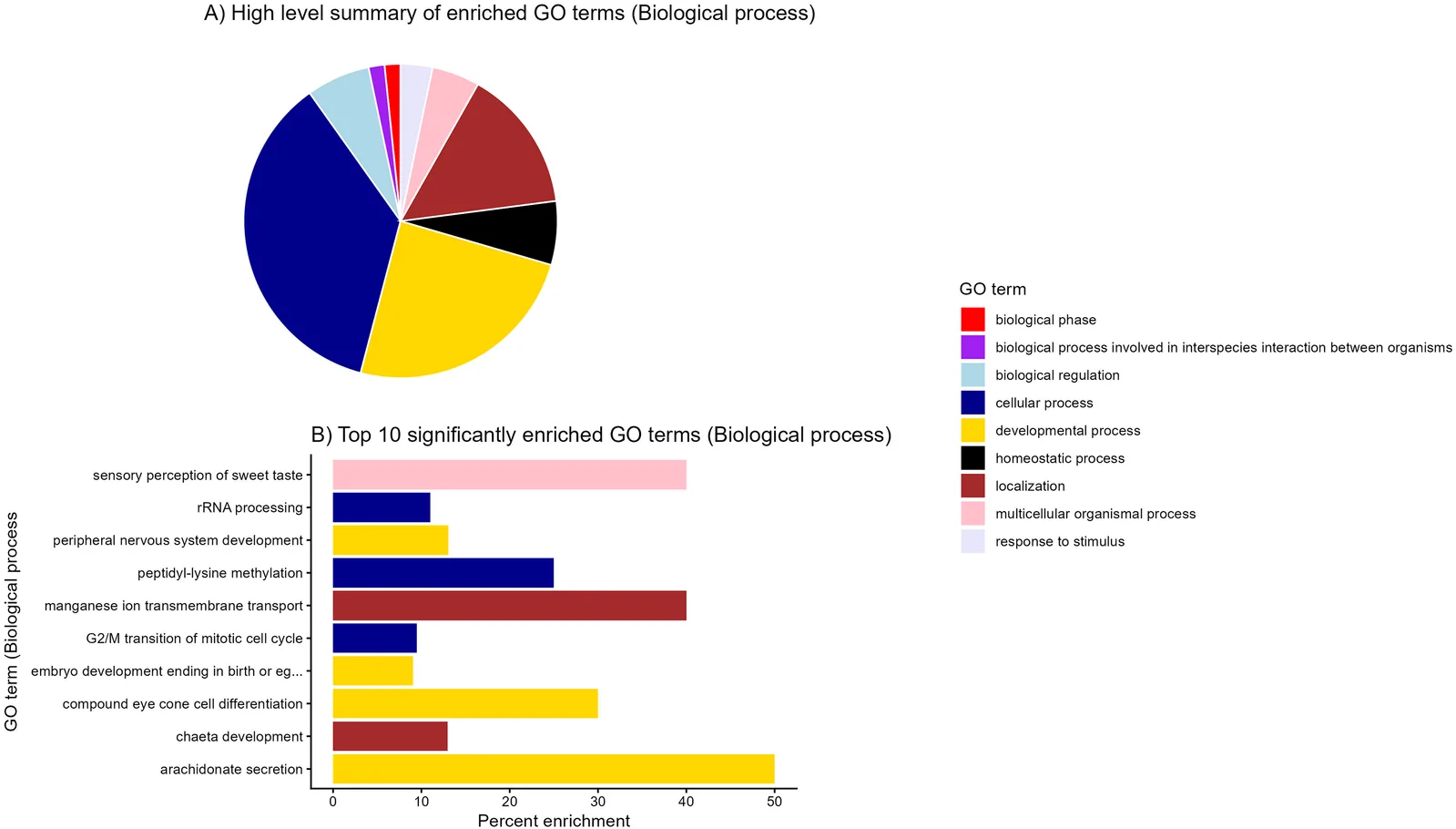
Enriched GO terms (biological process) in genes significantly upregulated on day 4 postinfection. The different colours in the pie chart represent different second-level terms of biological GO processes, and the areas the relative frequencies of the corresponding terms. The horizontal bars show the 10 most significantly enriched GO terms and the per cent of enrichment within the terms. The colours of the bar charts indicate which parent term (second level) they are associated with.

### (b) Experiment B: correlation between foraging activity and expression of *Malvolio*

#### (i) Foraging

Exposure to *B. bassiana* increased the overall foraging in the sub-colonies ($\chi^{2}$ = 24.680, d.f. = 1, *p* < 0.001). Regardless of treatment, sub-colonies overall preferred the standard food over the ROS food ($\chi^{2}$ = 458.111, d.f. = 1, *p* < 0.001). Exposure significantly affected foraging on the different food types (treatment × food type: $\chi^{2}$ = 25.754, d.f. = 1, *p* < 0.001) with exposed colonies foraging more compared to the control sub-colonies overall on both the standard food (*z* = −3.382, *p* < 0.001) and the ROS food (*z* = −6.323, *p* < 0.001). The clear preference towards standard food over ROS food was also significant for every individual day in both the control and exposed sub-colonies (supplementary material, table S5).

Time significantly affected overall foraging ($\chi^{2}$ = 140.333, d.f. = 12, *p* < 0.001) which was further affected by the infection treatment (treatment × day: $\chi^{2}$ = 95.76, d.f. = 12, *p* < 0.001). Exposed sub-colonies foraged significantly more on the standard food compared to the control sub-colonies on each of the days 5–9 (day 5: *z* = −4.444, *p* < 0.001; day 6: −4.261, *p* < 0.001; day 7: −4.408, *p* < 0.001; day 8: *z* = −5.867, *p* < 0.001; day 9: *z* = −2.404, *p* = 0.016), while on day 13, the control sub-colonies foraged significantly more on the standard food compared to the exposed sub-colonies (*z* = 2.090, *p* = 0.037). The exposed sub-colonies foraged significantly more on the ROS food compared to the control sub-colonies on days 4–10 (day 4: *z* = 2.324, *p* = 0.02; day 5: *z* = −3.209, *p* = 0.001; day 6: *z* = −2.929, *p* = 0.003; day 7: *z* = −3.539, *p* < 0.001; day 8: −3.029, *p* = 0.003; day 9: *z* = −2.632, *p* = 0.009; day 10: *z* = −2.667, *p* = 0.008) (figure 4A,B).

**Figure 4.**
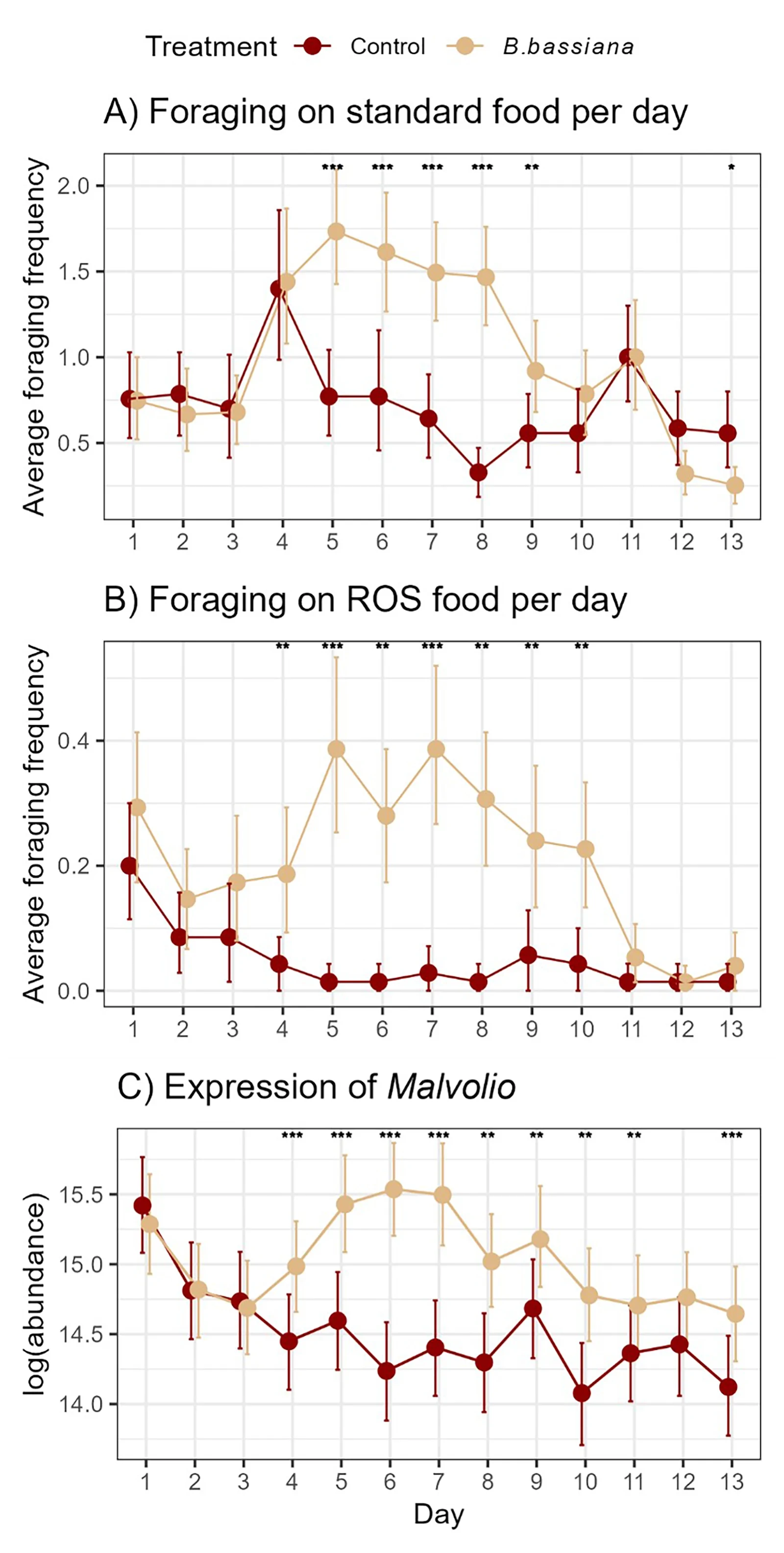
Daily dynamics of average foraging frequency. (A) The exposed colonies increased their foraging on the standard food on days 5–9 compared to the control colonies but foraged significantly less on it on day 13. (B) The foraging on ROS food started to differentiate on day 4, when exposed colonies increased their foraging, but foraging frequency decreased in control colonies. (C) Expression of *Malvolio* over time in the form of abundance of molecules amplified between control and exposed colonies. Exposed colonies showed a similar expression pattern to the foraging dynamics, with an increase and high expression after day 4 compared to control colonies, which showed a declining pattern in expression over the observation period. The peak in *Malvolio* expression in the exposed colonies was during days 5–7 but remained higher compared to the sham-treated colonies apart from day 12. This pattern of expression and foraging fits with the disease dynamics of *B. bassiana*. (A) and (B) post hoc pairwise comparison with Tukey's *p*-value adjustment, (C) MCMC.qpcr model results, ***p < 0.001, **p < 0.01, *p < 0.05. Whiskers indicate 95% confidence intervals; sample size *n* = 29 colonies.

#### (ii) Gene expression

We found significant temporal patterns of differential expression of *Malvolio* owing to exposure to the fungal pathogen (figure 4C). The exposed sub-colonies showed significantly higher expression on days 4–11 and on day 13 compared to the control sub-colonies (day 4: pMCMC = 0.012; day 5: pMCMC < 0.001; day 6: pMCMC < 0.001; day 7: pMCMC < 0.001; day 8: pMCMC = 0.008; day 9: pMCMC = 0.004; day 10: pMCMC = 0.004; day 11: pMCMC = 0.01; day 13: pMCMC < 0.001) (supplementary material, table S6).

## 4. Discussion

Here we show that *L. platythorax* ants medicate themselves when infected with the fungal pathogen *B. bassiana* by altering their preference to ROS-containing food during the acute phase of the disease. Feeding on ROS-containing foods was associated with improved survival, as colonies without access to a medicinal diet suffered from significant mortality caused by the pathogen. We identified *Malvolio* as a gene of interest in self-medication, as it was significantly upregulated during the acute phase of disease, at which time an increased dietary preference for ROS-containing food was clear. *Malvolio* affects the sensory perception of sweet taste in insects [19–21], and in our experiment, the expression pattern in infected *L. platythorax* correlates with the changes in foraging behaviour, suggesting that dietary self-medication could be influenced by infection-linked changes in sensory pathways.

The change in preference towards the ROS food between exposed and control sub-colonies is similar to what has been observed in self-medication studies with *F. fusca* against the same fungal pathogen in similar settings [8,9]. The exposed *L. platythorax* sub-colonies continuously foraged on the ROS food throughout the experiment, while the control sub-colonies barely interacted with it after the first few days, and the difference was clear during the acute infection phase (days 4–10). The alteration of foraging behaviour was more pronounced towards the ROS food than the standard food, which could mean that any changes in how the ants perceive dietary choices during infection are more attuned to the properties of the ROS food. Furthermore, consumption of ROS food was associated with increased survival of infected sub-colonies, as exposure to the pathogen no longer caused significant mortality. As H_2_O_2_ has detrimental effects on both healthy ants and *B. bassiana* [8], and we demonstrate that it is deliberately consumed upon infection, we can confirm self-medication in *L. platythorax* for the first time.

Exposure to *B. bassiana* caused significant changes in gene expression in the ants, and the small overlap of genes between the different time points shows that different genes are active in different stages of the infection. During the early stage of infection, we found several downregulated genes in processes related to metabolism as well as the detoxification of pesticides. Detoxification processes and metabolism are considered to be part of a trade-off with the function of immune genes in insects [50], which could imply that the immune systems of the ants are active, even if no genes directly linked to immunity were upregulated at this time. As the disease progressed to the acute phase, processes, such as the production of arachidonic acid, which is an anti-inflammatory response related to immunity, were enriched for in significantly upregulated genes, as were several metal transport processes, which are central in insect–microbe interactions [51] as well as neurotransmission [19].

Further, in our differential gene expression analyses, sensory perception of sweet taste emerged as another enriched process of particular interest, with the gene *Malvolio* as the primary driver, significantly upregulated in subpopulations experiencing pathogen infections. In a designated experiment (experiment B), *Malvolio* showed temporal expression patterns which correlate with changes in the foraging patterns of the exposed sub-colonies. We observed a similar timeline of significantly increased expression starting around day 4 and maintained in the infected colonies until around day 10, when the expression would decrease again. This temporal pattern is similar to what we see in foraging on the ROS food, which was significantly higher during days 4–10, consistent with a link between the expression of *Malvolio* and the shift towards foraging on the ROS food. Foraging on the standard food was also divergent during days 5–9, which falls into the period of significant overexpression of *Malvolio*, implying that foraging overall might be influenced by the gene activity.

The *Malvolio* gene is homologous and partially functionally complementary to the NRAMP (natural resistance-associated macrophage protein) gene family in vertebrates, which function as divalent metal ion transporters and are associated with immune and stress responses [20,47,52,53]. During pathogen infection, it is argued that NRAMPs prevent infection through local transfer of metal ions, limiting the amount of ions available for pathogens to grow within the hosts [49,54], which is likely to also be the function of *Malvolio* in insects. However, the activity of *Malvolio* in neuronal circuits has also been shown to lead to changes in feeding behaviours in *Drosophila* by influencing their responsiveness to sugar [19,20]. In honeybees, the expression levels of *Malvolio* influence the transition from nurse honeybees to foragers, as well as the decision to forage on pollen or sugar-rich nectar [21]. Compounds with toxic or medicinal properties are often described as bitter [4,5,8,9,18], meaning that a change in responsiveness to sweet tastes could further influence the perception of bitter-tasting diets, as these tastes are connected in insects [16]. Previous work on tiger moth caterpillars has shown that endoparasite infections alter the responsiveness to gustatory cells specific for pyrrolizidine alkaloids that they use for self-medication but also to other bitter deterrent compounds with medicinal potential, further highlighting a potential connection with taste perception and self-medication [18]. While the exact mechanism of how *Malvolio* affects the sensory perception in ants is still unknown, our results clearly show that the expression of the gene correlates with alterations in foraging decision-making to include medicinal compounds and strongly suggests that it could play a central role in self-medication. Therefore, our results warrant further investigation into understanding the relationship between *Malvolio* and self-medication behaviours and how pathogen infection influences sensory pathways in a way which increases the probability of self-medication.

The increasing evidence of self-medication behaviours in animals makes it evident that it is an important strategy for animals against the ubiquitous threat of pathogens. Uncovering the underlying processes to what drives this behaviour is essential to understanding how self-medication functions and how it has evolved. Owing to its interplay between immunity, taste perception and behavioural changes, *Malvolio* makes for an interesting candidate gene to further study the relationship between immune responses and behaviours and whether it has a causal influence in self-medication responses.

## Supplementary Material



## Data Availability

The raw RNA sequencing data were deposited to NCBI GenBank under the BioProject PRJNA1535060. The data files and scripts for analysis are publicly available in Zenodo [55]. Supplementary material is available online [56].
