# Supplementary material for: Infection-induced changes in gene expression linked to foraging choices in self-medicating Lasius platythorax ants
Source: Proc Biol Sci. 2026 Nov 11;293(2082):20262357. doi: 10.1098/rspb.2026.2357 (PMC13751121; doi:10.1098/rspb.2026.2357)
Supplement: Supplementary file 1 [file rspb.2026.2357.zip › rspb.2026.2357/rspb20262357_si_001.docx]

# Infection induced changes in gene expression linked to foraging choices in self-medicating *Lasius platythorax* ants

*Jason Rissanen^1,2^, Christoph Hahn^1^, Heikki Helanterä^2,3^ & Dalial Freitak^1,2^

^1^Institute of Biology, University of Graz, Graz 8010, Styria, Austria.

^2^Tvärminne Zoological Station, University of Helsinki, Hanko 10900, Finland

^3^Ecology and Genetics Research Unit, University of Oulu, Oulu 90014, Finland

Published in: Proceedings of the Royal Society B. DOI: 10.1098/rspb

# Electronic supplementary material:

In this supplementary material, we provide the full analysis tables for the enrichment analysis (biological processes) of the differentially expressed genes (tables 1 – 4), the full tabled results for pairwise analysis output for foraging on different food types on each day (experiment b) (table 5), and the full output of the MCMC.qpcr analysis of *Malvolio* and *Actin* (housekeeping gene) between control and infected colonies (table 6)

Table 1 GO terms enriched with downregulated genes on day 4 post exposure to Beauveria bassiana (topGO output).

Table 2 GO terms enriched with upregulated genes on day 4 post exposure to Beauveria bassiana. topGO output.

Table 3 GO terms enriched with downregulated genes on day 2 post exposure to Beauveria bassiana. topGO output.

Table 4 GO terms enriched with upregulated genes on day 2 post exposure to Beauveria bassiana. topGO output.

Table 5 Full table of pairwise comparisons between food types (Standard food / ROS food) within the infection treatment on each day.

Table 6 Full analysis output of MCMC.qpcr analysis of Malvolio and Actin expression between control and colonies exposed to Beauveria bassiana (TreatmentF). Significant results highlighted.

Table 7 Forward and reverse primer sequences for the general Malvolio gene (MalG) and the Actin housekeeping gene.
